# Supplementary material for: Trafficking of the telomerase RNA using a novel genetic approach
Source: PLoS One. 2025 Apr 2;20(4):e0313178. doi: 10.1371/journal.pone.0313178 (PMC11964246; doi:10.1371/journal.pone.0313178)
Supplement: S1 Table — (DOCX) [file pone.0313178.s001.docx]

**Supplementary Table 1.** Primer sequences and uses.

| Primers | Sequence (5’ - 3’) | Use/Description | Annealing Temperature (°C ) |
| --- | --- | --- | --- |
| PL1 | GGTGGACAATTTCGGTAGG | Left Flank Forward Primer (TER) | 63 |
| PL2 | GCAAGAGGTGAATACCGG | Left Flank Reverse Primer (TER) | 63 |
| PR3 | GCATGTTCACTAAGCCCC | Right Flank Forward Primer (TER) | 64 |
| PR4 | CTGGAGCAGGCTTTTGAG | Right Flank Reverse Primer (TER) and Integration Check TER locus specific reverse primer (TER) used with IC+pyrG-TERKO2ADW | 63 |
| PpyrG5 | CCGGTATTCACCTCTTGCGATTTCAGTAACGTTAAGTGGAT | 2 in 1 Left and pyrG fragment fusion primer (TER) | 75 |
| PpyrG6 | GGGGCTTAGTGAACATGCGACAGAAGATGATATTGAAGGAGC | 2 in 1 Right and pyrG fragment fusion primer (TER) | 77 |
| Pnested7 | CTCAGC TTCTGGAATGTCAG | Linear Knockout Construct Forward Nested Primer (TER) | 63 |
| Pnested8 | GCTAGCCTTGAGTATGGGG | Linear Knockout Construct Reverse Nested Primer (TER) | 65 |
| AS5FF-1 | CCCAGGAGCCTTACTTATG | Left Flank Forward Primer (TERT) | 62 |
| AS5FR-2 | GGTCGCTGAACGGAGAC | Left Flank Reverse Primer (TERT) | 66 |
| AS3FF-3 | GAATGGCCAACTCAATGAAG | Right Flank Forward Primer (TERT) | 61 |
| AS3FR-4 | GTCGGTGTAGACAAGAAG | Right Flank Reverse Primer (TERT) | 59 |
| AS5FRRPGF-5 | GTCTCCGTTCAGCGACCGATTTCAGTAACGTTAAGTGGAT | 2 in 1 Left and pyrG fragment fusion primer (TERT) and Integration Check pyrG specific forward primer (TERT) | 76 |
| AS3FFRPGR-6 | CTTCATTGAGTTGGCCATTCGACAGAAGATGATATTGAAGGAGC | 2 in 1 Right and pyrG fragment fusion primer (TERT) | 75 |
| BPNf | CAGATGGACTGTGGATTG | Linear Knockout Construct Forward Nested Primer (TERT) | 60 |
| ASNestedR | CTGATATCTAGGGCAGGC | Linear Knockout Construct Reverse Nested Primer (TERT) | 62 |
| IC+pyrG-TERKO2ADW | GCAATTGATTTGGTTGGGTC | Integration Check pyrG specific forward primer (TER) | 62 |
| BPF-IC+TER | GCTGCTATAGGGAAGATCCAC | Integration Check TER specific forward primer (TER) | 65 |
| FH_TNF_2 | GACTGCGCTAAGGATTCTGG | Integration Check TER locus specific reverse primer (TER); Must be used with BPF-IC+TER | 66 |
| LDBPR-IC | CGGTGTAGACAAGAAGAGTG | Integration Check TERT locus specific reverse primer (TERT) | 62 |
| BPF-IC+TERT | CGAGGACAGCCAGATTATG | Integration Check TERT specific forward primer | 62 |
