# Supplementary material for: Trafficking of the telomerase RNA using a novel genetic approach
Source: PLoS One. 2025 Apr 2;20(4):e0313178. doi: 10.1371/journal.pone.0313178 (PMC11964246; doi:10.1371/journal.pone.0313178)
Supplement: S2 Table — (DOCX) [file pone.0313178.s002.docx]

**Supplementary Table 2.** Media recipes and uses

| Solutions | Ingredients | Use/Description |
| --- | --- | --- |
| Yeast Media (1L): YAG, YAGUU, YGUU, YAG + 1M Sucrose, YAG +1M Sucrose top agar | 5g yeast extract | Used to transform, grow hyphae, and perform the heterokaryon test |
|  | 10g Glucose |  |
|  | 1.2g MgSO_4_ |  |
|  | 1mL Pyridoxine (0.5 mg/mL) |  |
|  | 1mL Hutner’s trace elements |  |
|  | 1.12g Uracil (omit for selective plates) |  |
|  | 1.2g Uridine (omit for selective plates) |  |
|  | 15g Agar (omit for liquid YGUU media) (half for top agar) |  |
|  | 342g Sucrose (omit for YAG, YAGUU, YGUU) |  |
|  | Make up to 1000ml with nanopure H_2_O |  |
| Malt Media (1L): MAG, MAGUU, MAGUU Top Agar | 20g Malt Extract | Used to grow conidia for harvesting and purify transformants |
|  | 10g Glucose |  |
|  | 2g Bactopeptone |  |
|  | 1mL Pyridoxine (0.5 mg/mL) |  |
|  | 1mL Hutner’s trace elements |  |
|  | 1mL 4-Aminobenzoic Acid (PABA) |  |
|  | 1.12g Uracil (omit for selective plates) |  |
|  | 1.2g Uridine (omit for selective plates) |  |
|  | 20g Agar (half for top agar) |  |
|  | Make up to 1000ml with nanopure H_2_O |  |
| Solution 1 | 0.8M Ammonium Sulfate (NH_4_)_2_SO_4_  100mM Citric Acid C_6_H_8_O_7_ | Used in the Lytic Solution |
|  | pH to 6 with KOH |  |
|  | Adjust final volume to 500mL with nanopure H_2_O |  |
|  | Autoclave and score at 4°C |  |
| Solution 2 | 5g Yeast Extract | Used in the Lytic Solution |
|  | 2% Sucrose |  |
|  | 20 mM Magnesium Sulfate MgSO_4_●7H_2_O |  |
|  | 1mL Trace elements |  |
|  | Adjust final volume to 500mL with nanopure H_2_O |  |
|  | Autoclave and store at 4°C |  |
| Solution 3 | 0.4M Ammonium Sulfate (NH_4_)_2_SO_4_ | Used to wash protoplasts after incubation in the lytic solution |
|  | 1% Sucrose |  |
|  | 50mM Citric Acid C_6_H_8_O_7_ |  |
|  | pH to 6 with KOH |  |
|  | Adjust final volume to 500mL with nanopure H_2_O |  |
|  | Autoclave at 10 psi for 15 minutes |  |
| Solution 4 | 25% PEG 8000 (Sigma-Aldrich) | Used during transformation when introducing the knockout construct to the protoplasts. Has polyethylene glycol (PEG) to facilitate transformation. |
|  | 100mM Calcium Chloride CaCl_2_●2H_2_O |  |
|  | 0.6M Potassium Chloride KCl |  |
|  | 10mM Tris-Cl [pH 7.5] (BioRad) |  |
|  | Adjust final volume to 100mL with nanopure H_2_O |  |
|  | Heat until PEG is dissolved and then filter sterilize into 15mL aliquots |  |
|  | Store at -20°C |  |
| Solution 5 | 0.6M Potassium Chloride KCl | Used to store protoplasts for up to 48 hours |
|  | 50mM Calcium Chloride CaCl_2_●2H_2_O |  |
|  | 10mM MOPS ((3-(N-morpholino)propanesulfonic acid) |  |
|  | pH to 6.0 with KOH |  |
|  | Adjust final volume to 50ml with nanopure H_2_O |  |
|  | Filter sterilize and store at 4°C |  |
| Lytic Solution | 12.5mL Solution 1 | Used to enzymatically degrade the cell wall of germinating conidia |
|  | 12.5mL Solution 2 |  |
|  | 50mg BSA |  |
|  | 50mg Vinoflow FCE (Gusmer Enterprises) |  |
| 1x fixative/permeation solution | 50mL of 37% formaldehyde | Used to fix the conidia and make them more permeable |
|  | 50mL of 500mM potassium phosphate buffer |  |
|  | 2mL of triton X-100 |  |
