# Supplementary material for: Trafficking of the telomerase RNA using a novel genetic approach
Source: PLoS One. 2025 Apr 2;20(4):e0313178. doi: 10.1371/journal.pone.0313178 (PMC11964246; doi:10.1371/journal.pone.0313178)
Supplement: S1 Fig — (PDF) [file pone.0313178.s003.pdf]

Supplementary Figure 1

(from 1-642 bp)

1/8/2025 4:27:25 PM

TER flanking with A. fumigatus pyrG (5973 bp)

```
BspDI                                                                                   SacI
ClaI                                                                                Eco53kI
GGTGCTGGTATTGCGATGGTTAATCGATGGTTAATCGTGTAGCTGGTTGAGGAATTGAGATGGTGAGGGGTCGGAAGATGGCGGCGGGGAGCTCAAAGCTATTTATC
CCACGACCATAACGCTACCAATTAGCTACCAATTAGCACATCGACCAACTCCTTAACTCTACCACTCCCCAGCCTTCTACCGCCGCCCCTCGAGTTTCGATAAATAG
Left intergenic
10 20 30 40 50 60 70 80 90 100

                                                  PshAI
                                         TatI        KflI          NcoI
CCGGCAGCCCCGCACTGAGAAGAAGCCTTGAGGCTGTACTTATTCGGGACCCGAGTCTCCTTCCATGGTCCAACGTGGTGCAACTATTGTGCCTGAAATTTTACCGA
GGCCGTCGGGGCGTGACTCTTCTTCGGAACTCCGACATGAATAAGCCCTGGGCTCAGAGGAAGGTACCAGGTTGCACCACGTTGATAACACGGACTTTAAAATGGCT
Left intergenic
110 120 130 140 150 160 170 180 190 200 210

GTGCGGCCCAACTAATAGAGATGTGTTTAGTCGACAGGACATGCTCGTCTTCCAAGGGTCAATGACAGAGACCGGTGTTTCTCCCAAGGTGCGACATACCACGGGTC
CACGCCGGGTTGATTATCTCTACACAAATCAGCTGTCCTGTACGAGCAGAAGGTTCCCAGTTACTGTCTCTGGCCACAAAGAGGGTTCCACGCTGTATGGTGCCCAG
Left intergenic
220 230 240 250 260 270 280 290 300 310 320

                                                                                                        SpeI
TTTAGTATATCTTTACTTGAACTTGAAGCAGGACCAGCCACTCTCAGCGGATAGAATGGTATTGTCCCTATCTTGAAACTGGAGTGCAATTCGCACTAGTCACAGAC
AAATCATATAGAAATGAACTTGAACTTCGTCCTGGTCGGTGAGAGTCGCCTATCTTACCATAACAGGGATAGAACTTTGACCTCACGTTAAGCGTGATCAGTGTCTG
Left intergenic
330 340 350 360 370 380 390 400 410 420

                                                            BsaHI                                        XbaI
TAAGCCCGGATTCAACGGCAAACGCTCAACGGCAAACGCTCAACTGCACAAGCTCGCTGGCGTCACGACCTCTTCAGTATGAACCTCGGTGACATCTTTAGGTCTAG
ATTCGGGCCTAAGTTGCCGTTTGCGAGTTGCCGTTTGCGAGTTGACGTGTTCGAGCGACCGCAGTGCTGGAGAAGTCATACTTGGAGCCACTGTAGAAATCCAGATC
Left intergenic
430 440 450 460 470 480 490 500 510 520 530

BspDI                                                                                  BstBI
ClaI                                                                               EcoRI         AseI
ACGAAATCGATTGAAGGTACTCGGTTTAAGCGGACGATCAGGATGCCTCCAGGGAGGATCATGGTTCCCAGAGCAAAACGGGAATTCGAATCTCAGGACCCATTAAT
TGCTTTAGCTAACTTCCATGAGCCAAATTCGCCTGCTAGTCCTACGGAGGTCCCTCCTAGTACCAAGGGTCTCGTTTTGCCCTTAAGCTTAGAGTCCTGGGTAATTA
Left intergenic
540 550 560 570 580 590 600 610 620 630 640
```

BspMI
BfuAI XcmI

CTTGGTGAGAGCTTGCCAACGATGAGTGACAAGCCTGGCAACAACAGACGAGGCGACGACCTGCAGCGCGGGCCAACAAGTGCTTGGACCGGTGGCTTGCGACTCGA
GAACCACTCTCGAACGGTTGCTACTCACTGTTCGGACCGTTGTTGTCTGCTCCGCTGCTGGACGTCGCGCCCGGTTGTTCACGAACCTGGCCACCGAACGCTGAGCT

Left intergenic

650 660 670 680 690 700 710 720 730 740

BciVI

ATGGGGCTGATGACGCACGTTAGTATCCCGAGGGGTGCATTGCAAGACGAGAATCATCGGCATAGCCTATCTAATGAACCAGTAAGCTTTGACACCTTCAATTGTGG
TACCCCGACTACTGCGTGCAATCATAGGGCTCCCCACGTAACGTTCTGCTCTTAGTAGCCGTATCGGATAGATTACTTGGTCATTCGAAACTGTGGAAGTTAACACC

Left intergenic

750 760 770 780 790 800 810 820 830 840 850

BsrGI
TatI NmeAIII

GGTGGACATCGGCAAAAGTTTAAGGACTGCAAAGAAACTGGTTGGTAAGTTCGCTGTACAGTGTCAGCAAGGGGGTTATTTGGCGGGTGACTGTCAGTCTAAGACAT
CCACCTGTAGCCGTTTTCAAATTCCTGACGTTTCTTTGACCAACCATTCAAGCGACATGTCACAGTCGTTCCCCCAATAAACCGCCCACTGACAGTCAGATTCTGTA

Left intergenic

860 870 880 890 900 910 920 930 940 950 960

CGTTCGTTGAAAGCGGCTCGGCTCTGATTTGGCAGAGGGCAGCATCTCTTCCATGACAGGGTAGGTAGCATTATAGGGGTGCTCAGTGAGAGAAGCAATTATCATGG
GCAAGCAACTTTCGCCGAGCCGAGACTAAACCGTCTCCCGTCGTAGAGAAGGTACTGTCCCATCCATCGTAATATCCCCACGAGTCACTCTCTTCGTTAATAGTACC

Left intergenic

970 980 990 1,000 1,010 1,020 1,030 1,040 1,050 1,060 1,070

AflII BsmI

AACGAATCTGCTTAAGAGAGTGTACCATCTGCTGCTCTATCTGTTTTTGCTGCAAGGGTACTCGACCTACACCGAGGTTTGGAATGCTGGTCGAGTCTGAAGCACAT
TTGCTTAGACGAATTCTCTCACATGGTAGACGACGAGATAGACAAAAACGACGTTCCCATGAGCTGGATGTGGCTCCAAACCTTACGACCAGCTCAGACTTCGTGTA

Left intergenic

1,080 1,090 1,100 1,110 1,120 1,130 1,140 1,150 1,160 1,170

BstAPI BstBI

CTTGCAGCTGAAACAAATGCCGGAAAGGATGGTCACGTAACTAATGATGGAGAGGGGCCTGGTGGGAAGATGAAGCGAAGTGATGTTCGAAGTTTTCCCATTTGTTC
GAACGTCGACTTTGTTTACGGCCTTTCCTACCAGTGCATTGATTACTACCTCTCCCCGGACCACCCTTCTACTTCGCTTCACTACAAGCTTCAAAAGGGTAAACAAG

Left intergenic

1,180 1,190 1,200 1,210 1,220 1,230 1,240 1,250 1,260 1,270 1,280

PmlI

TATGTCTTTATTTTTATATCATTATTTAATCGTACCAAATCGCGTTCATGCCGAATCCTTGCTCTGGAATATCCACACGTGCCACGCGAGAAAGATTCTCGTCACGG
ATACAGAAATAAAAATATAGTAATAAATTAGCATGGTTTAGCGCAAGTACGGCTTAGGAACGAGACCTTATAGGTGTGCACGGTGCGCTCTTTCTAAGAGCAGTGCC

Left intergenic

1,290 1,300 1,310 1,320 1,330 1,340 1,350 1,360 1,370 1,380 1,390

HaeII BstXI

AATCTGTAAATCTCGACGAAGCCCAGCTGGTCATCGCAGAGCGCCAGCCACTCGTCACTCCAGTCGCATGGGCTCACAGCGTTGGAGTGGCCGCCGCTGGTGGAAGT
TTAGACATTTAGAGCTGCTTCGGGTCGACCAGTAGCGTCTCGCGGTCGGTGAGCAGTGAGGTCAGCGTACCCGAGTGTCGCAACCTCACCGGCGGCGACCACCTTCA

Left intergenic

1,400 1,410 1,420 1,430 1,440 1,450 1,460 1,470 1,480 1,490

HaeII BglI

AGGGTGGATAAACAGCTGGCGCTCGATGTTGCCGTTCGGGCCCAGCTTGAAAGCGGTAATGTAACCGGTCACATCAAAGTGGTTGCTGCGGGTAGTTGCGAAAAGAT
TCCCACCTATTTGTCGACCGCGAGCTACAACGGCAAGCCCGGGTCGAACTTTCGCCATTACATTGGCCAGTGTAGTTTCACCAACGACGCCCATCAACGCTTTTCTA

Left intergenic

1,500 1,510 1,520 1,530 1,540 1,550 1,560 1,570 1,580 1,590 1,600

SacII StuI NruI

ATTCACCGCTGCGAGTAGTGAAAGTGACATCACCGCGGTACTTGTTGCGGGGGGGAAGGCCTATAAATGTCAGATCAAAGCAGGATTGTCGGTTCGCGAGACATACC
TAAGTGGCGACGCTCATCACTTTCACTGTAGTGGCGCCATGAACAACGCCCCCCCTTCCGGATATTTACAGTCTAGTTTCGTCCTAACAGCCAAGCGCTCTGTATGG

Left intergenic

1,610 1,620 1,630 1,640 1,650 1,660 1,670 1,680 1,690 1,700 1,710

AloI

AGGAGGAAGTAAAGGATATGTGATGTGCGTAAAGACAGGCTTGTGGGTTCGCTCGTCAATGACGTAGACGGCCAAGCGGTTGCCAGCTTCCATCAGAGCATAGAGGT
TCCTCCTTCATTTCCTATACACTACACGCATTTCTGTCCGAACACCCAAGCGAGCAGTTACTGCATCTGCCGGTTCGCCAACGGTCGAAGGTAGTCTCGTATCTCCA

Left intergenic

1,720 1,730 1,740 1,750 1,760 1,770 1,780 1,790 1,800 1,810

EcoNI

ACTTGCCGCTGGGGTGCATTTCAACCCAGCGAGGGTGATCGCCAGGGTCAGGGGCCTCAAGACAATCGACGAGACTCAGTTCTCCTGTCTTAGGGTCTTTGAGATGG
TGAACGGCGACCCCACGTAAAGTTGGGTCGCTCCCACTAGCGGTCCCAGTCCCCGGAGTTCTGTTAGCTGCTCTGAGTCAAGAGGACAGAATCCCAGAAACTCTACC

Left intergenic

1,820 1,830 1,840 1,850 1,860 1,870 1,880 1,890 1,900 1,910 1,920

BglII

GTCCAGATCTTGTTGGCCTGCAAATCCGCAGAGTATAAGTAGGTTTCGGTAGGGTCAAACACCATGCCGTGAATACCGGTGTTCGGCTCGTACTCGTAGTTCTGGAT
CAGGTCTAGAACAACCGGACGTTTAGGCGTCTCATATTCATCCAAAGCCATCCCAGTTTGTGGTACGGCACTTATGGCCACAAGCCGAGCATGAGCATCAAGACCTA

Left intergenic

1,930 1,940 1,950 1,960 1,970 1,980 1,990 2,000 2,010 2,020 2,030

MscI
PsrI PshAI

GTTCTTGGCCAGACGACCGTCCGATTCTACTGAGAACACATTGCCGAATCCAGCGTACTTATAGAACGGATTTCCATAAACGTTATAGGGAGGTTTGCGGGCTGCGA
CAAGAACCGGTCTGCTGGCAGGCTAAGATGACTCTTGTGTAACGGCTTAGGTCGCATGAATATCTTGCCTAAAGGTATTTGCAATATCCCTCCAAACGCCCGACGCT

Left intergenic

2,040 2,050 2,060 2,070 2,080 2,090 2,100 2,110 2,120 2,130 2,140

BlpI BsiWI

GTACGAAGATTGCGCGTGTGTTTGTGTCTTCATTGGCGGCCATAGCTGTGAAAGAGAAAGTCAGTCAAGACTGCTCAGCTGCGCGGAAAGCAGGAGTAGACGTACGA
CATGCTTCTAACGCGCACACAAACACAGAAGTAACCGCCGGTATCGACACTTTCTCTTTCAGTCAGTTCTGACGAGTCGACGCGCCTTTCGTCCTCATCTGCATGCT

Left intergenic

2,150 2,160 2,170 2,180 2,190 2,200 2,210 2,220 2,230 2,240

PflMI XmnI

TGCCCAGCAACTGGATGAGATACTTGGTGGACAATTTCGGTAGGGCTGTTGACTGCGAAGCTATTCCACTTCTTCATGGCAGCACCATAGATGGTTTTTTTGTCATG
ACGGGTCGTTGACCTACTCTATGAACCACCTGTTAAAGCCATCCCGACAACTGACGCTTCGATAAGGTGAAGAAGTACCGTCGTGGTATCTACCAAAAAAACAGTAC

PL1

Left intergenic

2,250 2,260 2,270 2,280 2,290 2,300 2,310 2,320 2,330 2,340 2,350

BlpI

CTGCTTTGGTTAGACACGCCAATGAAGTACCAAATGAAGAACATACAGAGAAAGTCATCCATGAGATCGGCTCAGCTTCTGGAATGTCAGTCTTCTTAACCAATTCC
GACGAAACCAATCTGTGCGGTTACTTCATGGTTTACTTCTTGTATGTCTCTTTCAGTAGGTACTCTAGCCGAGTCGAAGACCTTACAGTCAGAAGAATTGGTTAAGG

Pnested7

Left intergenic

2,360 2,370 2,380 2,390 2,400 2,410 2,420 2,430 2,440 2,450 2,460

AGAGTCAAAGCCTCGTCATCGAAAGCAACTGTATAGATGCGGCCTGGACGGGTCCAAGTGCCCACCATCAAATGATGCTTCATCCTGACTGACTGAGTGACTGGTCG
TCTCAGTTTCGGAGCAGTAGCTTTCGTTGACATATCTACGCCGGACCTGCCCAGGTTCACGGGTGGTAGTTTACTACGAAGTAGGACTGACTGACTCACTGACCAGC

Left intergenic

2,470 2,480 2,490 2,500 2,510 2,520 2,530 2,540 2,550 2,560

Tth111I
EcoRV PflFI BciVI

CAATTGACGGAGATATCGAGGAGACGAAGTCGTCACACGCCACATCAGGAGCAGTAACAGAAGAAGAGGAGGGGAGGTATTCAAATGGATACTGAGGTTAAGAACGG
GTTAACTGCCTCTATAGCTCCTCTGCTTCAGCAGTGTGCGGTGTAGTCCTCGTCATTGTCTTCTTCTCCTCCCCTCCATAAGTTTACCTATGACTCCAATTCTTGCC

Left intergenic

2,570 2,580 2,590 2,600 2,610 2,620 2,630 2,640 2,650 2,660 2,670

Bsu36I SphI BsrBI

CAATCAATCACGGCAGCGGGCCTTGAGTGCCTGAGGCATGCTTCCCCAGAAGCCCCGCAAAGAGCGGATGAAGAAATTGCCGCTAGAACATCCCAACTCTCGAATCA
GTTAGTTAGTGCCGTCGCCCGGAACTCACGGACTCCGTACGAAGGGGTCTTCGGGGCGTTTCTCGCCTACTTCTTTAACGGCGATCTTGTAGGGTTGAGAGCTTAGT

Left intergenic

2,680 2,690 2,700 2,710 2,720 2,730 2,740 2,750 2,760 2,770 2,780

Tth111I
PflFI EagI

GGAACAACTGCAGATTGTTTTCGTCATCATTGTTGTAATGCCGGAGACCAAGTCTGGAGAGGTACGCGGCAATTGTAGAGCCTATCGAAGCCAGCCAGCCTGCGGCC
CCTTGTTGACGTCTAACAAAAGCAGTAGTAACAACATTACGGCCTCTGGTTCAGACCTCTCCATGCGCCGTTAACATCTCGGATAGCTTCGGTCGGTCGGACGCCGG

Left intergenic

2,790 2,800 2,810 2,820 2,830 2,840 2,850 2,860 2,870 2,880

PfoI
NdeI BamHI

GAAGCATATGTAAGATACAGCGGTCGAGTAGGCACGGCAGTAGGCGAGGCGGTTGAGGTTCAGTCAATCATCGGCAGTCAGCTGCCTGGATCCCGGAAAGCGGTTTG
CTTCGTATACATTCTATGTCGCCAGCTCATCCGTGCCGTCATCCGCTCCGCCAACTCCAAGTCAGTTAGTAGCCGTCAGTCGACGGACCTAGGGCCTTTCGCCAAAC

Left intergenic

2,890 2,900 2,910 2,920 2,930 2,940 2,950 2,960 2,970 2,980 2,990

MluI

CTGCGGCAGAATCCCAAGCTTGAGGCGCAGATCCCTGCATCACATGACTACGCGTCGCGTTTACCAAAGCAGGCCGTGTTGTAACGTCCTCTCTCTTCCTCTTCCCA
GACGCCGTCTTAGGGTTCGAACTCCGCGTCTAGGGACGTAGTGTACTGATGCGCAGCGCAAATGGTTTCGTCCGGCACAACATTGCAGGAGAGAGAAGGAGAAGGGT

Left intergenic

3,000 3,010 3,020 3,030 3,040 3,050 3,060 3,070 3,080 3,090 3,100

BtsαI BstZ17I
BsrBI BglII BtsI SnaBI

CTCATCCATCAGCTTTCGCATCGCTCCCGCTCAACCAATTTTCTGAGAAGATCTCGCTTGCCACTGCTCATGGTATCGCTAATACGTATACTCGTCAAACAACACGT
GAGTAGGTAGTCGAAAGCGTAGCGAGGGCGAGTTGGTTAAAAGACTCTTCTAGAGCGAACGGTGACGAGTACCATAGCGATTATGCATATGAGCAGTTTGTTGTGCA

TER gene

3,110 3,120 3,130 3,140 3,150 3,160 3,170 3,180 3,190 3,200 3,210

TTACCGCGCGAAATCTGTCTTTCCGTGTCCGAAACCCCGCCGGTATTCACCTCTTGCGATTTCAGTAACGTTAAGTGGATCAAAGACATGATCTCTTACTGAGAGTT
AATGGCGCGCTTTAGACAGAAAGGCACAGGCTTTGGGGCGGCCATAAGTGGAGAACGCTAAAGTCATTGCAATTCACCTAGTTTCTGTACTAGAGAATGACTCTCAA

ppyrG5
PL2
TER gene pyrG Af

3,220 3,230 3,240 3,250 3,260 3,270 3,280 3,290 3,300 3,310

ATTCTGTGTCTGACGAAATATGTTGTGTATATATATATATGTACGTTAAAAGTTCCGTGGAGTTACCAGTGATTGACCAATGTTTTATCTTCTACAGTTCTGCCTGT
TAAGACACAGACTGCTTTATACAACACATATATATATATACATGCAATTTTCAAGGCACCTCAATGGTCACTAACTGGTTACAAAATAGAAGATGTCAAGACGGACA

pyrG Af

3,320 3,330 3,340 3,350 3,360 3,370 3,380 3,390 3,400 3,410 3,420

AleI
EciI

CTACCCCATTCTAGCTGTACCTGACTACAGAATAGTTTAATTGTGGTTGACCCCACAGTCGGAGGCGGAGGAATACAGCACCGATGTGGCCTGTCTCCATCCAGATT
GATGGGGTAAGATCGACATGGACTGATGTCTTATCAAATTAACACCAACTGGGGTGTCAGCCTCCGCCTCCTTATGTCGTGGCTACACCGGACAGAGGTAGGTCTAA

pyrG Af

3,430 3,440 3,450 3,460 3,470 3,480 3,490 3,500 3,510 3,520 3,530

DraI SspI

GGCACGCAATTTTTACACGCGGAAAAGATCGAGATAGAGTACGACTTTAAATTTAGTCCCCGGCGGCTTCTATTTTAGAATATTTGAGATTTGATTCTCAAGCAATT
CCGTGCGTTAAAAATGTGCGCCTTTTCTAGCTCTATCTCATGCTGAAATTTAAATCAGGGGCCGCCGAAGATAAAATCTTATAAACTCTAAACTAAGAGTTCGTTAA

pyrG Af

3,540 3,550 3,560 3,570 3,580 3,590 3,600 3,610 3,620 3,630

BstEII NmeAIII

GATTTGGTTGGGTCACCCTCAATTGGATAATATACCTCATTGCTCGGCTACTTCAACTCATCAATCACCGTCATACCCCGCATATAACCCTCCATTCCCACGATGTC
CTAAACCAACCCAGTGGGAGTTAACCTATTATATGGAGTAACGAGCCGATGAAGTTGAGTAGTTAGTGGCAGTATGGGGCGTATATTGGGAGGTAAGGGTGCTACAG

IC+p...DW
pyrG Af

3,640 3,650 3,660 3,670 3,680 3,690 3,700 3,710 3,720 3,730 3,740

PaeR7I
PspXI
XhoI
TliI
BstXI

GTCCAAGTCGCAATTGACTTACGGTGCTCGAGCCAGCAAGCACCCCAATCCTCTGGCAAAGAGACTTTTTGAGATTGCCGAAGCAAAGAAGACAAACGTTACCGTCT
CAGGTTCAGCGTTAACTGAATGCCACGAGCTCGGTCGTTCGTGGGGTTAGGAGACCGTTTCTCTGAAAAACTCTAACGGCTTCGTTTCTTCTGTTTGCAATGGCAGA

pyrG Af

3,750 3,760 3,770 3,780 3,790 3,800 3,810 3,820 3,830 3,840 3,850

PfoI BsiWI BamHI NdeI

CTGCTGATGTGACGACAACCCGAGAACTCCTGGACCTCGCTGACCGTACGGAAGCTGTTGGATCCAATACATATGCCGTCTAGCAATGGACTAATCAACTTTTGATG
GACGACTACACTGCTGTTGGGCTCTTGAGGACCTGGAGCGACTGGCATGCCTTCGACAACCTAGGTTATGTATACGGCAGATCGTTACCTGATTAGTTGAAAACTAC

pyrG Af

3,860 3,870 3,880 3,890 3,900 3,910 3,920 3,930 3,940 3,950

ATACAGGTCTCGGTCCCTACATCGCCGTCATCAAGACACACATCGACATCCTCACCGATTTCAGCGTCGACACTATCAATGGCCTGAATGTGCTGGCTCAAAAGCAC
TATGTCCAGAGCCAGGGATGTAGCGGCAGTAGTTCTGTGTGTAGCTGTAGGAGTGGCTAAAGTCGCAGCTGTGATAGTTACCGGACTTACACGACCGAGTTTTCGTG

pyrG Af

3,960 3,970 3,980 3,990 4,000 4,010 4,020 4,030 4,040 4,050 4,060

AleI

AACTTTTTGATCTTCGAGGACCGCAAATTCATCGACATCGGCAATACCGTCCAGAAGCAATACCACGGCGGTGCTCTGAGGATCTCCGAATGGGCCCACATTATCAA
TTGAAAAACTAGAAGCTCCTGGCGTTTAAGTAGCTGTAGCCGTTATGGCAGGTCTTCGTTATGGTGCCGCCACGAGACTCCTAGAGGCTTACCCGGGTGTAATAGTT

pyrG Af

4,070 4,080 4,090 4,100 4,110 4,120 4,130 4,140 4,150 4,160 4,170

FspI EcoNI

CTGCAGCGTTCTCCCTGGCGAGGGCATCGTCGAGGCTCTGGCCCAGACCGCATCTGCGCAAGACTTCCCCTATGGTCCTGAGAGAGGACTGTTGGTCCTGGCAGAGA
GACGTCGCAAGAGGGACCGCTCCCGTAGCAGCTCCGAGACCGGGTCTGGCGTAGACGCGTTCTGAAGGGGATACCAGGACTCTCTCCTGACAACCAGGACCGTCTCT

pyrG Af

4,180 4,190 4,200 4,210 4,220 4,230 4,240 4,250 4,260 4,270 4,280

XcmI BstZ17I

TGACCTCCAAAGGATCGCTGGCTACGGGCGAGTATACCAAGGCATCGGTTGACTACGCTCGCAAATACAAGAACTTCGTTATGGGTTTCGTGTCGACGCGGGCCCTG
ACTGGAGGTTTCCTAGCGACCGATGCCCGCTCATATGGTTCCGTAGCCAACTGATGCGAGCGTTTATGTTCTTGAAGCAATACCCAAAGCACAGCTGCGCCCGGGAC

pyrG Af

4,290 4,300 4,310 4,320 4,330 4,340 4,350 4,360 4,370 4,380

AloI

ACGGAAGTGCAGTCGGATGTGTCTTCAGCCTCGGAGGATGAAGATTTCGTGGTCTTCACGACGGGTGTGAACCTCTCTTCCAAAGGAGATAAGCTTGGACAGCAATA
TGCCTTCACGTCAGCCTACACAGAAGTCGGAGCCTCCTACTTCTAAAGCACCAGAAGTGCTGCCCACACTTGGAGAGAAGGTTTCCTCTATTCGAACCTGTCGTTAT

pyrG Af

4,390 4,400 4,410 4,420 4,430 4,440 4,450 4,460 4,470 4,480 4,490

KpnI
Acc65I
BanI BanI

CCAGACTCCTGCATCGGCTATTGGACGCGGTGCCGACTTTATCATCGCCGGTCGAGGCATCTACGCTGCTCCCGACCCGGTTGAAGCTGCACAGCGGTACCAGAAAG
GGTCTGAGGACGTAGCCGATAACCTGCGCCACGGCTGAAATAGTAGCGGCCAGCTCCGTAGATGCGACGAGGGCTGGGCCAACTTCGACGTGTCGCCATGGTCTTTC

pyrG Af

4,500 4,510 4,520 4,530 4,540 4,550 4,560 4,570 4,580 4,590 4,600

MscI BspHI BarI

AAGGCTGGGAAGCTTATATGGCCAGAGTATGCGGCAAGTCATGATTTCCTCTTGGAGCAAAAGTGTAGTGCCAGTACGAGTGTTGTGGAGGAAGGCTGCATACATTG
TTCCGACCCTTCGAATATACCGGTCTCATACGCCGTTCAGTACTAAAGGAGAACCTCGTTTTCACATCACGGTCATGCTCACAACACCTCCTTCCGACGTATGTAAC

pyrG Af

4,610 4,620 4,630 4,640 4,650 4,660 4,670 4,680 4,690 4,700

SacI
Eco53kI EciI

TGCCTGTCATTAAACGATGAGCTCGTCCGTATTGGCCCCTGTAATGCCATGTTTTCCGCCCCCAATCGTCAAGGTTTTCCCTTTGTTAGATTCCTACCAGTCATCTA
ACGGACAGTAATTTGCTACTCGAGCAGGCATAACCGGGGACATTACGGTACAAAAGGCGGGGGTTAGCAGTTCCAAAAGGGAAACAATCTAAGGATGGTCAGTAGAT

pyrG Af

4,710 4,720 4,730 4,740 4,750 4,760 4,770 4,780 4,790 4,800 4,810

Bsu36I

GCAAGTGAGGTAAGCTTTGCCAGAAACGCCAAGGCTTTATCTATGTAGTCGATAAGCAAAGTGGACTGATAGCTTAATATGGAAGGTCCCTCAGGACAAGTCGACCT
CGTTCACTCCATTCGAAACGGTCTTTGCGGTTCCGAAATAGATACATCAGCTATTCGTTTCACCTGACTATCGAATTATACCTTCCAGGGAGTCCTGTTCAGCTGGA

pyrG Af

4,820 4,830 4,840 4,850 4,860 4,870 4,880 4,890 4,900 4,910 4,920

GTGCAGAAGAGATAACAGCTTGGCATCACGCATCAGTGCCTCCTCTCAGACAGGCTCCTTCAATATCATCTTCTGTCGCATGTTCACTAAGCCCCACCGTCCAGACC
CACGTCTTCTCTATTGTCGAACCGTAGTGCGTAGTCACGGAGGAGAGTCTGTCCGAGGAAGTTATAGTAGAAGACAGCGTACAAGTGATTCGGGGTGGCAGGTCTGG

TER gene
PR3
PpyrG6
pyrG Af

4,930 4,940 4,950 4,960 4,970 4,980 4,990 5,000 5,010 5,020

BssSαI
BssSI

CTGCGATACTCTTCCTTCAAACTCAACGACCCTTTCCATCCATTCCGTGCCTCTCTACTGAAGGTGCTGGAACCAACGATGGCGGGACCTCTCTCGTGTGAGGCTCA
GACGCTATGAGAAGGAAGTTTGAGTTGCTGGGAAAGGTAGGTAAGGCACGGAGAGATGACTTCCACGACCTTGGTTGCTACCGCCCTGGAGAGAGCACACTCCGAGT

TER gene

5,030 5,040 5,050 5,060 5,070 5,080 5,090 5,100 5,110 5,120 5,130

```
AAGTTTAGGCTTGTCTGCTGTTTGTTCGGTGAAAGTTCCTGTAGGTTTGACACTCATGGGTAAACTGATCGGGTTTTGTGTCTCTGGAGAAAGAATCGGAGTGTGTT
TTCAAATCCGAACAGACGACAAACAAGCCACTTTCAAGGACATCCAAACTGTGAGTACCCATTTGACTAGCCCAAAACACAGAGACCTCTTTCTTAGCCTCACACAA
```
TER gene

5,140 5,150 5,160 5,170 5,180 5,190 5,200 5,210 5,220 5,230 5,240

```
CTGCTGGTGTGGGCTGGTGAATGGGCCGTTTCCCTTCGGGGGACGGTCCCCCCCTTTTTTTATACGTCTCCCCCTCTTGTTCAGTGGCTCTGAGTGTTAAGTGGCTA
GACGACCACACCCGACCACTTACCCGGCAAAGGGAAGCCCCCTGCCAGGGGGGGAAAAAAATATGCAGAGGGGGAGAACAAGTCACCGAGACTCACAATTCACCGAT
```
TER gene

Right intergenic

5,250 5,260 5,270 5,280 5,290 5,300 5,310 5,320 5,330 5,340 5,350

PaeR7I
XhoI
TliI
SspI

```
ACTGCATTGCAGGGTTGATGATTTATTGGGTTGATTACCCTGGTTTTAATATTCTCGAGCCTTGACTCTTGCAGCAACACCTAGCCTCATAACATAAACAACTTGAT
TGACGTAACGTCCCAACTACTAAATAACCCAACTAATGGGACCAAAATTATAAGAGCTCGGAACTGAGAACGTCGTTGTGGATCGGAGTATTGTATTTGTTGAACTA
```
Right intergenic

5,360 5,370 5,380 5,390 5,400 5,410 5,420 5,430 5,440 5,450

PsrI BsmI

```
AGGAAGCGGACTTGCCATTGGGCCAAAGGGTACGAAGCGTACTGGTATCATGTGTTCGGAGCTGGTGAAGATTATGGATGAGGTATAAAAAAAAGTCGTACTTTGCA
TCCTTCGCCTGAACGGTAACCCGGTTTCCCATGCTTCGCATGACCATAGTACACAAGCCTCGACCACTTCTAATACCTACTCCATATTTTTTTTCAGCATGAAACGT
```
Right intergenic

5,460 5,470 5,480 5,490 5,500 5,510 5,520 5,530 5,540 5,550 5,560

```
TTCCAGTTGGAAACTAGCAAGTTAAGTTAGGGCCGGACTCTTAACTATGGGAAGCGTCACCAATCGTTAAGCGATTTGAAAGATAACACCTTCTACTGTCGTATGAG
AAGGTCAACCTTTGATCGTTCAATTCAATCCCGGCCTGAGAATTGATACCCTTCGCAGTGGTTAGCAATTCGCTAAACTTTCTATTGTGGAAGATGACAGCATACTC
```
Right intergenic

5,570 5,580 5,590 5,600 5,610 5,620 5,630 5,640 5,650 5,660 5,670

EcoRV

```
ATTTGACGGAGATATCCAAATCTCGCGTAATCGCAACAGGTTGTGAGTGTATCTCTGATGCCAGATTCCAAGAGCAAATCAGGACTCGCAGAGACAGCTGAAGTTAC
TAAACTGCCTCTATAGGTTTAGAGCGCATTAGCGTTGTCCAACACTCACATAGAGACTACGGTCTAAGGTTCTCGTTTAGTCCTGAGCGTCTCTGTCGACTTCAATG
```
Right intergenic

5,680 5,690 5,700 5,710 5,720 5,730 5,740 5,750 5,760 5,770

```
AACTGCTTTTTTCTATGTCTATCCTACCCTAAACCAAGCCAGAAGCAGTAATGCTAATTGGTATATCGACTCAAACCAAATCAAAGCGGAATACAAGTCATAATATG
TTGACGAAAAAAGATACAGATAGGATGGGATTTGGTTCGGTCTTCGTCATTACGATTAACCATATAGCTGAGTTTGGTTTAGTTTCGCCTTATGTTCAGTATTATAC
```
Right intergenic

5,780 5,790 5,800 5,810 5,820 5,830 5,840 5,850 5,860 5,870 5,880

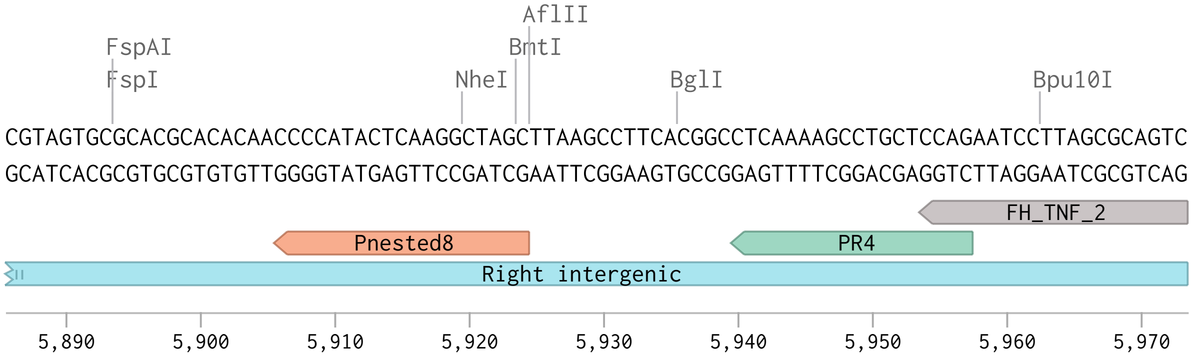
AflII
FspAI
BmtI
FspI
NheI
BglI
Bpu10I
CGTAGTGCGCACGCACACAACCCCATACTCAAGGCTAGCTTAAGCCTTCACGGCCTCAAAAGCCTGCTCCAGAATCCTTAGCGCAGTC
GCATCACGCGTGCGTGTGTTGGGGTATGAGTTCCGATCGAATTCGGAAGTGCCGGAGTTTTCGGACGAGGTCTTAGGAATCGCGTCAG
FH_TNF_2
Pnested8
PR4
Right intergenic
5,890
5,900
5,910
5,920
5,930
5,940
5,950
5,960
5,970
