# Supplementary figures and images for: Trafficking of the telomerase RNA using a novel genetic approach
Source: PLoS One. 2025 Apr 2;20(4):e0313178. doi: 10.1371/journal.pone.0313178 (PMC11964246; doi:10.1371/journal.pone.0313178)

## Slide 1
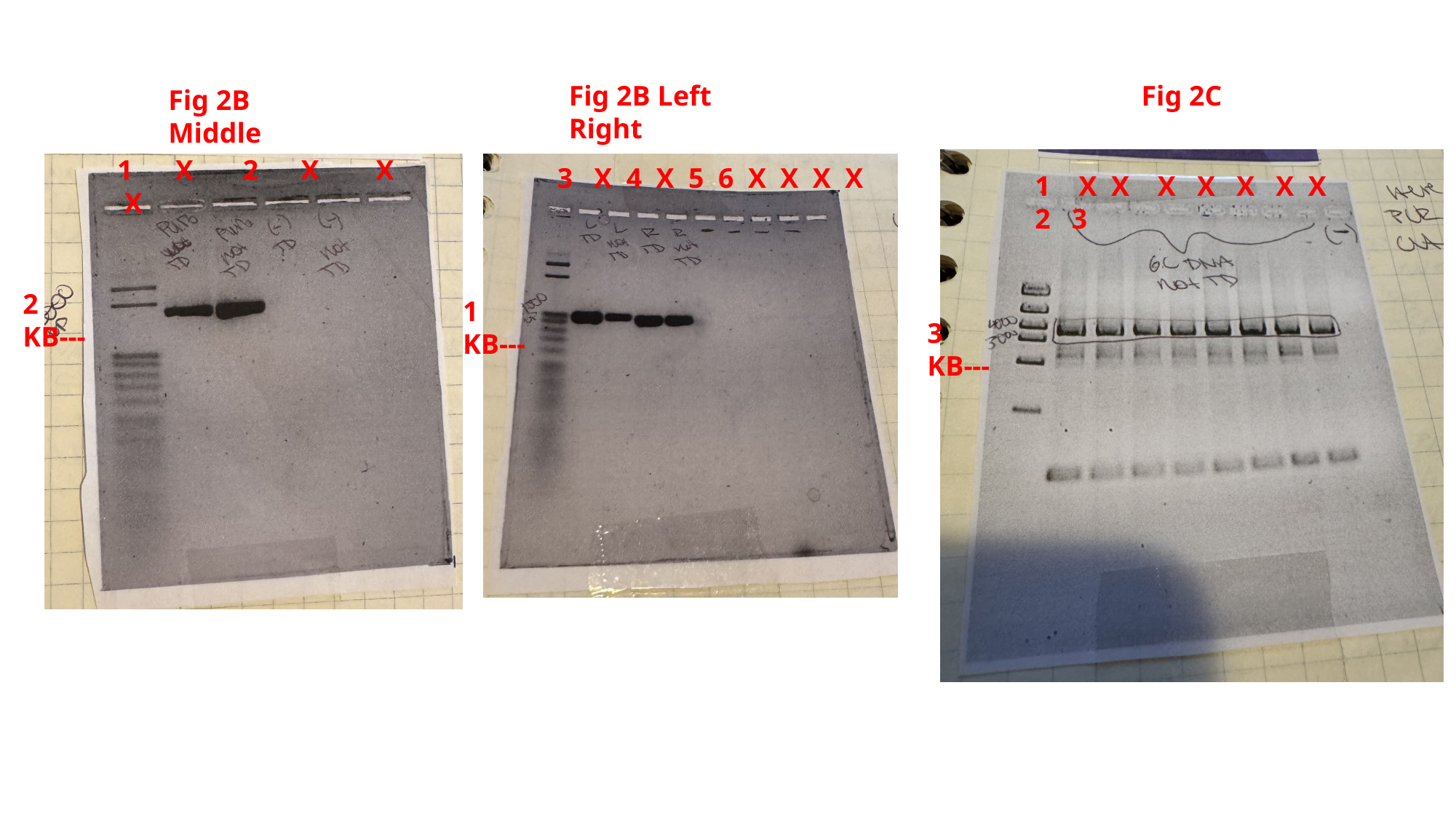

Fig 2B Left Right
Fig 2C
Fig 2B Middle
1 X 2 X X X
3 X 4 X 5 6 X X X X
1 X X X X X X X 2 3
2 KB---
1 KB---
3 KB---

## Slide 2
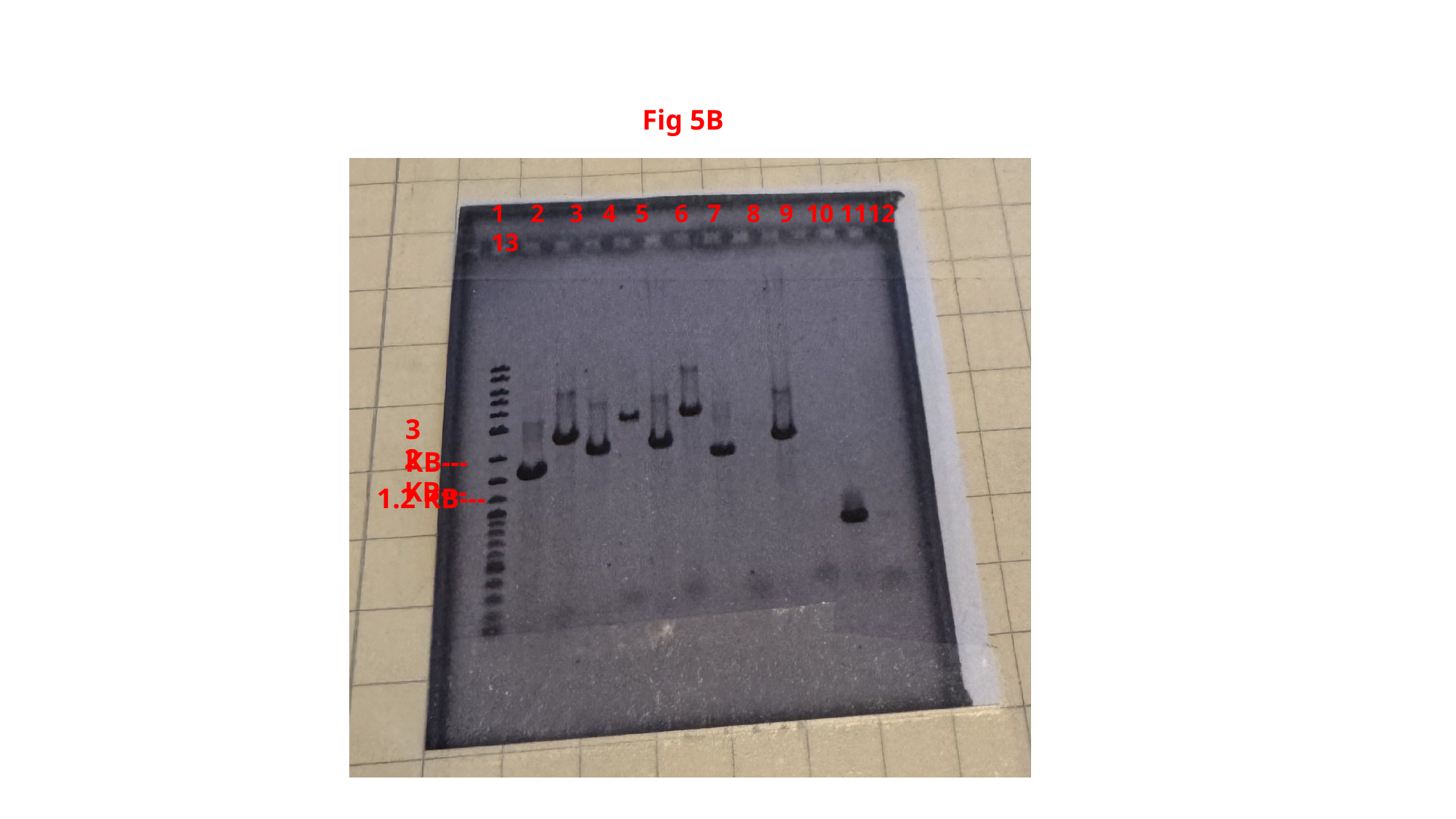

Fig 5B
1 2 3 4 5 6 7 8 9 10 1112 13
3 KB---
2 KB---
1.2 KB---

Supplement: S2 Fig — (PPTX) [file pone.0313178.s004.pptx]
